# Aluminum-activated root malate and citrate exudation is independent of NIP1;2-facilitated root-cell-wall aluminum removal in *Arabidopsis*

**DOI:** 10.1080/15592324.2017.1422469

**Published:** 2018-01-17

**Authors:** Yuqi Wang, Yanfei Cai, Yu Cao, Jiping Liu

**Affiliations:** a Key Laboratory for Water Quality and Conservation of the Pearl River Delta, Ministry of Education, School of Environmental Science and Engineering, Guangzhou University, Guangzhou, Guangdong, China; b Robert W. Holley Center, US Department of Agriculture, Agricultural Research Service, Cornell University, Ithaca, NY, USA; c College of Natural Resources and Environment, South China Agricultural University, Guangzhou, Guangdong, China

**Keywords:** Acid soils, aluminum toxicity, aluminum tolerance, organic acids, aquaporin, ALMT1, MATE, NIP1;2

## Abstract

In *Arabidopsis*, aluminum (Al) exclusion from the root is mainly facilitated by Al-activated root malate and citrate exudation through the ALMT1 malate transporter and the MATE citrate transporter, respectively. However, the nature of an internal Al tolerance mechanism remains largely unknown. In a recent study, we showed that NIP1;2 facilitates Al-malate transport from the root cell wall into the root symplasm and subsequent root-to-shoot translocation and thus NIP1;2 plays key roles in Al detoxification and internal tolerance in *Arabidopsis*. We discovered that the NIP1;2-mediated Al removal from the root cell wall requires a functional ALMT1-mediated malate exudation system, which allows the formation of an Al-malate complex in the root cell wall. Thus, a coordinated function between the exclusion and the internal resistance mechanisms, linked by the ALMT1-mediated root malate exudation and the NIP1;2-mediated Al uptake system, is critical for Al resistance in *Arabidopsis*.

In acid soils (pH < 5), toxic aluminum (Al) ions, Al^3+^, are released from aluminosilicate clays into the soil solution, causing root growth inhibition and limited nutrient and water uptake by plants.^1^ As acid soils are widely distributed around the world, Al toxicity is a major constraint that limits crop yields on acid soils worldwide.^1-4^

Plants have evolved several Al resistant mechanisms, including (1) a well characterized exclusion mechanism, through which plants release organic acids (OAs), e.g., malate and citrate, or other organic ligands from the root into the rhizosphere, which prevents toxic Al^3+^ ions from entering into the root cells; (2) a less well characterized internal tolerance mechanism through which the toxic cytosolic Al in the root is sequestered to the vacuole of the root cell and/or translocated to the shoot for further sequestration in the leaf vacuole.^2^^,^^3^^,^^5^

Over the past decade, key components involved in the Al exclusion mechanism have been identified in different plant species.^6^ A unique feature for the cloned Al resistance genes is that they commonly encode plasma-membrane (PM) localized malate or citrate transporters from the Al-activated malate transporter (ALMT) and the multidrug and toxic compound extrusion (MATE) families and facilitate Al-activated malate and citrate exudation, respectively, in wheat, sorghum, barley, maize and *Arabidopsis*.^7-13^ In *Arabidopsis thaliana*, transcriptional expression of *ALMT1* and *MATE* is controlled by a master zinc-finger transcription factor, STOP1.^11,14,15^ Recently we have identified several *stop1* suppressor mutants, which could help reveal the STOP1-mediated signaling pathways involved in low pH and Al resistance in *Arabidopsis*.^16^

In contrast, the internal Al resistance mechanism is less well characterized in plants. Recent studies identified OsNrat1 (Nramp aluminum transporter 1) as a putative Al transporter involved in the internal resistance mechanism in rice that lowers Al concentrations in the root cell wall.^17^^,^^18^

## Aluminum resistance in *Arabidopsis*

In *Arabidopsis*, the Al exclusion mechanism is mainly achieved by Al-activated malate and citrate exudation from the root.^8^^,^ ^11^ *ALMT1* is expressed in the root tip region and is responsible for a large amount of Al-activated malate releases from the root tip upon Al stress, while *MATE* plays a smaller but significant role in Al resistance, responsible for releases of a smaller amount of citrate from the mature root region.^12^

## Aluminum retained in the root cell wall is toxic to plants

**Figure 1.**
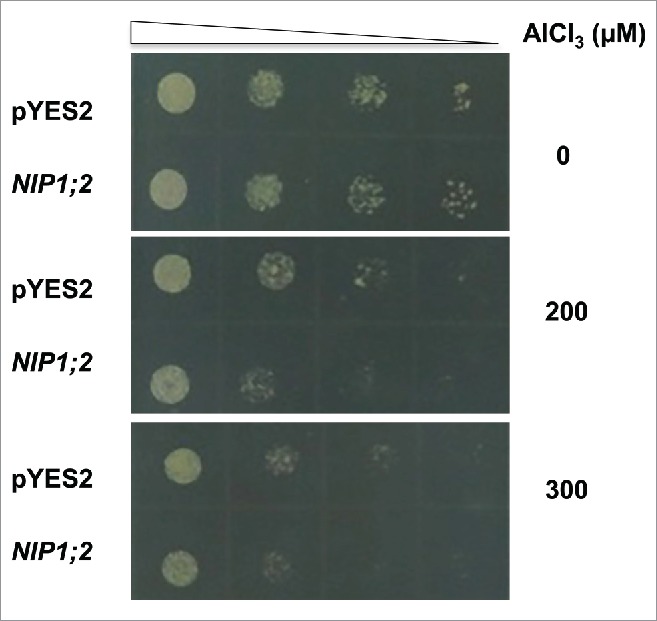
NIP1;2-expressing yeast cells are hyper-sensitive to Al stress. Yeast cells (BY4741) carrying an empty vector (pYES2) or a pYES2-NIP1;2 (NIP1;2) construct were cultured in a liquid SD-Ura medium to a stationary phase before collected by centrifugation and then washed 3 times with deionized water. Yeast cells then underwent four 10-fold serial dilutions with a low pH, low magnesium (LPM) medium (pH 4.2). Then, 5 μL of each dilution sample was spotted onto LPM plates containing 0, 200 or 300 μM AlCl3 supplied with 2% galactose for induction of the GAL promoter, 300 mM malate and buffered with 5 mM succinic acid at pH 4.2, and. The LPM plates were placed in a 30^o^C incubator for 3 d.

Increasing evidence has demonstrated that the root cell wall in the root tip region is a major target for Al toxicity in plants.^19^^,^^20^ Our recent studies indicated that even with a functional Al-activated OA exudation system that prevents Al from entering the root cell, the simple binding of Al and malate is not enough to provide full protection.^21^ The Al-malate complexes in the cell wall need to be removed from the root tip region to reach a higher level of Al resistance.^21^ We have demonstrated that NIP1;2, a PM-localized member of the nodolin 26-like intrinsic protein (NIP) subfamily of the aquaporin (AQP) superfamily, functions as an Al-malate (Al-Mal) transport that facilitates Al-Mal transport from the root cell wall into the root cytosol in the root tip region, and subsequent root-to-shoot Al translocation in *Arabidopsis*.^21^ *NIP1;2* is expressed in the root tip and this expression is enhanced by Al stress.^21^ When heterologously expressed in *Saccharomyces cerevisiae* (BY4741 strain), NIP1;2 facilitated Al-Mal transport across the PM.^21^ Under a control condition (-Al), no growth difference could be observed between a control yeast line transformed with an empty vector and an NIP1;2-expressing line (Fig. 1), suggesting that heterologously expressed NIP1;2 was not harmful for normal yeast growth. However, when supplied with 200 or 300 μM Al-malate, the *NIP1;2*-expressing yeast line displayed a hyper-sensitive phenotype to Al stress (Fig. 1). Such a growth inhibition can be explained by the NIP1;2-facilitated Al accumulation in the yeast cells.^21^

## Al-activated root organic acid exudation is independent of NIP1;2 function

In *Arabidopsis*, an *almt1* knockout (KO) mutation completely abolishes the ALMT1-mediated root malate exudation upon Al stress and thus the corresponding *almt1*-KO mutant is extremely hypersensitive to Al toxicity.^8^ In our recent study, we demonstrated that under Al stress, *almt1*-KO was also defective in removing Al from the root cell wall in spite of the fact that this mutant has a functional NIP1;2.^21^ Interestingly, when malate was externally supplied, the NIP1;2-mediated Al removal from the root cell wall was restored in *almt1*-KO, indicating that ALMT1-mediated root malate exudation is a prerequisite for the NIP1;2-mediated Al-Mal transport into the cytosol of the root cell in *Arabidopsis*.^21^

**Figure 2.**
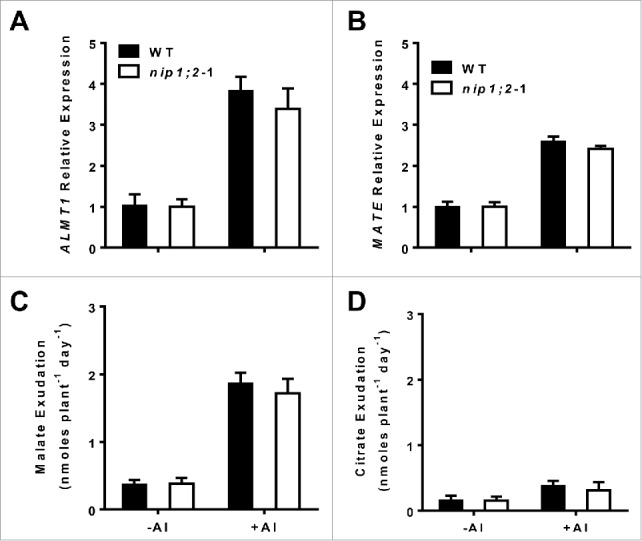
Patterns of transcriptional expression of *ALMT1* (A) and *MATE* (B) and Al-activated malate (C) and citrate (D) exudation from the root in the wild type and the *nip1;2* mutant. Here, 7-d-old seedlings were exposed to a hydroponic solution containing 30 μM AlCl^3^. Root samples were collected at 0, 6 h for qRT-PCR analysis. The *Arabidopsis* Ubiquitin gene (At1g31340) was used as an internal gene expression control. For root organic acid exudation assays, seedlings were treated with 0 or 30 μM AlCl^3^ for 24 h, then root exudation samples were collected for measuring malate and citrate contents. Values are means ± SD of three biological replicates.

To investigate functional relationships between OA releases and NIP1;2-mediated Al transport, patterns of *ALMT1* and *MATE* mRNA expression and root malate and citrate exudation were analyzed in the wild-type (WT) and *nip1;2* mutant backgrounds (Fig. 2). qRT-PCR analyses indicated that in WT, the transcriptional expression of both *ALMT1* and *MATE* was strongly induced by 24 h Al treatment (Fig. 2A, B). Al treatment also led to releases of a large amount of malate and a smaller amount of citrate from the root (Fig. 2C, D). These results are consistent with the previously reported.^11^^,^^12^ Compared with in the WT, the transcriptional expression of *ALMT1* and *MATE* (Fig. 2A, B) and the ALMT1-mediate malate and MATE-mediated citrate exudation (Fig. 2C, D) were slightly, but insignificantly, lower in the *nip1;2* mutant background, indicating that the *nip1;2* mutation does not significantly affect *ALMT1* and *MATE* gene expression and Al-activated malate and citrate exudation. In our recent study, we have demonstrated that MATE is not involved in the processes of the coordinated function between ALMT1 and NIP1;2.^21^ Taken together, our results suggest that ALMT1 is functionally epistatic to NIP1;2, but the ALMT1 function is independent of NIP1;2.

## Abbreviations

ALMT1: aluminum-activated malate transporter 1
Al-Mal: aluminum malate
AQP: aquaporin
KO: knockout
MATE: multidrug and toxic compound extrusion
Nrat1: Nramp aluminum transporter 1
NIP: nodulin 26-like intrinsic protein
OAs: organic acids
PM: plasma membrane
STOP1: sensitive to proton rhizotoxicity 1
WT: wild type
